# Identification of Candidate Genes for Alkaline Tolerance in Rice Seedlings Using BSA-Seq and RNA-Seq

**DOI:** 10.3390/genes17101162

**Published:** 2026-09-22

**Authors:** Bo Ma, Haoqiang Du, Xingyu Wang, Xiaolin Liu, Xiabing Sheng, Kefei Tan, Jifang Hu, Kai Liu, Rui Liu, Yixuan Ma, Xiayu Guo

**Affiliations:** 1 Qiqihar Branch of Heilongjiang Academy of Agricultural Sciences, Qiqihar 161006, China; 2 Northeast Branch of National Saline-Alkali-Tolerant Rice Technology Innovation Center, Harbin 150086, China; 3 National Center of Technology Innovation for Saline-Alkali Tolerant Rice in Sanya, Sanya 572019, China; 4 School of Economics and Management, Qiqihar University, Qiqihar 161006, China; 5 College of Life Sciences, Agriculture and Forestry, Qiqihar University, Qiqihar 161006, China

**Keywords:** rice, alkaline stress, BAS-Seq, RNA-Seq, ROS

## Abstract

Alkaline stress is one of the most critical limiting factors affecting high and stable rice yields. Therefore, analyzing the impact of alkaline stress on rice productivity and identifying salt- and alkali-tolerant genes are crucial for breeding rice varieties with strong stress resistance. In this study, the alkali-sensitive variety QJ74 and the alkali-tolerant variety QJ22 were used as parental lines. Under alkaline conditions (pH 9.0), 30 alkali-sensitive and 30 alkali-tolerant F_2_ individuals from the segregating population were selected for BSA-Seq. Transcriptomic analysis was performed on the parental lines under the same alkaline treatment. The results showed that under alkali stress, QJ22 exhibited significantly greater plant height and chlorophyll content, but significantly lower relative electrical conductivity and malondialdehyde content than QJ74, indicating less severe cell membrane damage. Meanwhile, QJ22 showed significantly higher proline content and superoxide dismutase and peroxidase activities, together with significantly lower Na^+^ and higher K^+^ contents compared with QJ74. Overall, QJ22 outperformed QJ74 in cell membrane stability, photosynthetic pigment maintenance, antioxidant enzyme activity, and osmotic regulation, demonstrating strong alkali tolerance. Based on these findings, under alkali stress (pH 9.0), 30 alkali-sensitive and 30 alkali-tolerant F_2_ individuals from the cross between the two parents were selected for BSA-Seq, and transcriptomic sequencing was performed on the parental lines under the same conditions. A combined BSA-Seq and RNA-Seq analysis identified five candidate genes (*Os10g0376200*, *Os10g0389000*, *Os10g0389500*, *Os10g0390500* and *Os10g0401000*). Subsequent *cis*-acting element analysis, phylogenetic analysis, and qRT-PCR validation revealed that *Os10g0389000* is a promising alkali stress-responsive candidate gene potentially contributing to the differential alkali tolerance between QJ74 and QJ22. This study integrated BSA-Seq and RNA-Seq to systematically identify candidate genes involved in the alkali stress response in rice, providing key genetic targets and a molecular basis for developing new alkali-tolerant varieties.

## 1. Introduction

Soil salinization and alkalization have become one of the main factors limiting crop growth, reducing yields by suppressing crop development and degrading soil health [1]. Driven by climate change, improper fertilization, and industrial and agricultural activities, the global area of saline alkali land has expanded steadily in recent years. In China, the total saline alkali land area has reached nearly 100 million mu and is still growing, posing a serious challenge to food security [2]. Among China’s major rice-growing regions, the saline alkali soils in the Northeast are characterized by high concentrations of carbonates and are rich in alkaline salts such as sodium bicarbonate (NaHCO_3_) and sodium carbonate (Na_2_CO_3_) [3]. Rice is somewhat sensitive to salinity, and the salt tolerance of its seedlings is a key factor determining final yield [4]. Therefore, identifying genes associated with salt and alkali tolerance in rice is of great theoretical and practical significance.

Salinity and alkali stress can inhibit plant growth and development through various mechanisms. Excessive Na^+^ in the soil can disrupt ion homeostasis in root cells, leading to ion toxicity and metabolic disorders. To adapt, plants must precisely regulate intracellular K^+^/Na^+^ balance through coordinated ion channel activities, thereby alleviating Na^+^ toxicity [5,6]. Upon detecting external stress, cell membrane receptors transduce the signals into intracellular Ca^2+^ signals. These calcium signals activate the SOS pathway, which promotes Na^+^ efflux from the cytoplasm and concurrently activates the vacuolar NHX1 transporter to sequester Na^+^ into the vacuole, thereby maintaining cytoplasmic ion homeostasis [6,7,8]. In addition, K^+^ not only contributes to osmoregulation and the maintenance of ionic balance but also stabilizes various physiological and biochemical processes, helping alleviate abiotic stresses [9]. Under saline alkali stress, a massive influx of Na^+^ triggers membrane depolarization, causing abnormal activation of outward-rectifying K^+^ channels and thus exacerbating K^+^ loss and membrane damage [10]. To counteract this stress, plants enhance extracellular K^+^ uptake by upregulating K^+^ transporters such as *HKT*, *HAK*, and *AKT* [11,12]. Meanwhile, plasma membrane H^+^-ATPase activity is also a key factor limiting K^+^ uptake [13]. In addition, upon stress perception, plasma membrane receptors transduce signals that trigger rapid changes in intracellular Ca^2+^ concentration, subsequently leading to the production of secondary messengers such as reactive oxygen species (ROS) and abscisic acid (ABA) [14,15,16]. These signals are transduced through a protein kinase cascade involving *MAPK* and *CDPK*, which ultimately activates downstream transcription factors to initiate the transcription of stress-responsive genes, thereby enabling plants to tolerate stress and repair damage [17,18,19]. In this signaling network, transcription factors play a central role in linking upstream signal perception to downstream gene expression, while also broadly regulating plant growth and development [20]. The *AP2/ERF* transcription factor *CaERF14*, induced by signaling molecules such as ABA and MeJA, activates downstream stress-responsive genes by binding to the GCC box, thereby enhancing salt and drought tolerance in pepper [21]. In rice, *OsMYB2* regulates genes encoding proline biosynthetic enzymes, LEA proteins, and ion transporters, enhancing osmotic adjustment and reducing membrane damage, thereby increasing abiotic stress tolerance [22].

Advances in high-throughput sequencing technologies and the dramatic reduction in next-generation sequencing (NGS) costs have rendered traditional gene mapping methods relatively time-consuming and labor-intensive. The integration of transcriptomic analysis (RNA-Seq) with BSA-Seq has therefore emerged as a rapid gene mapping approach, now widely used across diverse crops. Lv et al. [23] identified loci associated with chlorosis and antioxidant responses in soybean using BSA-Seq and RNA-Seq. Yan et al. [24] identified *Zm00001d048841* as the most likely candidate gene for short-ear syndrome in maize by integrating BSA-Seq and RNA-Seq. In another study, this combined approach was applied to Brassica napus, leading to the identification of multiple candidate genes associated with nitrogen use efficiency (NUtE) [25]. However, to date, the application of BSA-Seq combined with RNA-Seq for mapping genes involved in the alkali stress response of rice remains poorly explored. Using physiological experiments, BSA-Seq, and RNA-Seq, this study investigated differences in physiological parameters, gene expression, and pathway expression in rice under alkali stress. The aim is to identify genes involved in regulating rice salt and alkali tolerance, thereby providing a basis for gene discovery and molecular breeding for improved tolerance.

## 2. Materials and Methods

### 2.1. Plant Growth and Alkaline Stress Treatment

In this study, the rice varieties QJ22 (maternal parent) and Q74 (paternal parent) were selected as parents, and the F_1_ generation was obtained through hybridization. F_1_ plants were self-pollinated at the Sanya experimental station in Hainan to produce an F_2_ segregating population comprising 300 individual plants. Seeds were surface-sterilized with a 10% sodium hypochlorite solution for 30 min and then sown on culture medium for germination. Two weeks later, the seedlings were transplanted into Hoagland’s nutrient solution. At the three-leaf stage, seedlings with uniform growth were selected and transplanted into pots filled with soil, with one seedling per pot. The plants were then irrigated with a 75 mM NaHCO_3_ solution to maintain the soil pH at approximately 9.0. This treatment was continued for 14 consecutive days. Plant height was measured for each F_2_ plant, and from these, 30 plants exhibiting the most alkali-tolerant phenotype and 30 exhibiting the most alkali-sensitive phenotype were selected for sampling and used for BSA sequencing.

Simultaneously, seeds of the parental lines QJ22 and Q74 were surface-sterilized using the same procedure and grown hydroponically until the three-leaf stage. The seedlings were then transplanted into pots filled with soil and subjected to either normal conditions or alkaline stress (75 mM NaHCO_3_, pH 9.0), with three replicates per treatment. After 14 days, samples were collected for physiological analysis and RNA sequencing, with three replicates per treatment.

### 2.2. Measurement of Chlorophyll and Proline Content

Chlorophyll was extracted from 100 mg of fresh leaf tissue by grinding in 80% acetone. Absorbance was measured at 663 nm and 645 nm using a UV–Vis spectrophotometer (UV2400), and chlorophyll content was calculated [26].

Proline content in leaves was determined using a proline assay kit (Boxbio, Beijing, China, AKAM003M). Briefly, 0.1 g of leaf tissue was homogenized in 1 mL of extraction solution on an ice bath. The homogenate was incubated in a boiling water bath for 10 min and then centrifuged at 10,000× *g* for 10 min at room temperature. The supernatant was collected, cooled, and its absorbance was measured at 520 nm [27].

### 2.3. Measurement of Conductivity and MDA Content

Samples were rinsed with deionized water, blotted dry, and then placed in a test tube containing 25 mL of deionized water. After incubation at 25 °C for 1 h, the conductivity of the leachate was measured before and after salt treatment using a conductivity meter (recorded as a1 and a2, respectively). Finally, the samples were placed in a boiling water bath for 15 min, and the post-boiling conductivity (absolute conductivity; recorded as a_3_) was measured [28]. The relative conductivity (%) of each sample, an indicator of cell membrane permeability, was calculated using the following formula [29]:

Relative conductivity = (a_2_ − a_1_)/(a_3_ − a_1_) × 100

For MDA measurement, approximately 0.2 g of leaf tissue was homogenized in 1.5 mL of 10% trichloroacetic acid. After centrifugation, approximately 1 mL of supernatant was transferred to a clean 15-mL tube containing 0.25% thiobarbituric acid in 10% trichloroacetic acid. The sample was incubated at 100 °C for 30 min, cooled to room temperature, and centrifuged at 1× *g* for 15 min. The absorbance of the supernatant was measured at 450, 532, and 600 nm. MDA content was calculated using the following formula: 6.45 × (OD_532_ − OD_600_) − 0.559 × OD_450_ [30].

### 2.4. Determination of Antioxidant Enzyme Activity

A specific amount of plant tissue was weighed, and pre-chilled phosphate buffer (pH 7.4) was added at a ratio of 1:9 (*w*/*v*). The mixture was homogenized in an ice bath, centrifuged at 3500 rpm for 10 min at 4 °C, and the supernatant was collected for analysis. Superoxide dismutase (SOD) and peroxidase (POD) activities were measured using the corresponding assay kits (Boxbio; AKAO008M and AKAO005M, respectively). The protein concentration in the supernatant was determined by the Bradford method. Following the kit instructions, the absorbance was measured at 550 nm and 420 nm, respectively, and the specific activities of SOD and POD were calculated based on the protein concentration [31].

### 2.5. Measurement of Na^+^ and K^+^ Concentrations

For determination of Na^+^ and K^+^ contents, the collected samples were rinsed with deionized water, dried in paper bags at 80 °C to constant weight, and then weighed. The samples were then ashed in a muffle furnace at 575 °C for 9 h, digested in 0.1 N HCl for 4 h, and diluted 50-fold with 0.1 N HCl. The Na^+^ and K^+^ concentrations were subsequently determined by atomic absorption spectrophotometry [32].

### 2.6. BSA Sequencing and Candidate Region Mapping

After 14 days of alkali stress treatment, root samples from 30 alkali-tolerant and 30 alkali-sensitive plants were collected to form the two extreme pools. The samples were then submitted to BGI Shenzhen Co., Ltd. (Shenzhen, China) for BSA sequencing. Genomic DNA was extracted, and its integrity was assessed by agarose gel electrophoresis. DNA concentration and purity were evaluated using a NanoDrop spectrophotometer (Thermo Fisher Scientific, Waltham, MA, USA), and the concentration was accurately quantified with a Qubit fluorometer (Invitrogen, Carlsbad, CA, USA). Sequencing was performed on the Illumina HiSeq platform, and raw data were generated with an average coverage depth of 50× for both parental lines and the two mixed pools. Raw reads were quality-filtered to obtain clean reads. The clean reads were then aligned to the rice reference genome using BWA. Single-nucleotide variants (SNVs) were called and filtered using GATK4. Association analysis was performed using the Euclidean distance (ED), ΔSNP-index, and G-matrix methods [33]. Genomic regions detected by all three approaches were identified as candidate alkaline-tolerance regions.

### 2.7. Transcriptome Sequencing and Differential Analysis

Samples were collected from both parental lines following 14 days of normal or alkaline stress treatment, with three replicates per treatment. Total RNA was extracted using the RNA-easy Isolation Reagent kit, and cDNA libraries were constructed. The cDNA libraries were amplified by PCR, enriched, and the target fragments were recovered from a 2% agarose gel to ensure that only qualified libraries were used for transcriptomic analysis. Sequencing was performed on the Illumina platform by Hangzhou Kaitai Biotechnology Co., Ltd. (Hangzhou, China).

Raw sequencing data were filtered and quality-controlled using fastp [34] and fastqc [35]. The cleaned reads were then aligned to the rice reference genome with HISAT2 [36], and gene-level counts were quantified using featureCounts [37]. Gene expression levels were normalized using the median-of-ratios method implemented in DESeq2 [38] with the thresholds |log2FC| > 1 and *p* < 0.05, and enrichment analysis was performed with clusterProfiler [39].

### 2.8. Analysis of Promoter Cis-Acting Regulatory Elements

The rice reference genome was obtained from the Ensembl Plants database (https://plants.ensembl.org/index.html, accessed 29 July 2026), and the 2-kb promoter sequence upstream of each candidate gene was extracted. The promoter sequences were uploaded in FASTA format to the PlantCARE web server for online prediction (https://bioinformatics.psb.ugent.be/webtools/plantcare/html/, accessed 29 July 2026) [40], and the results were plotted using the ggplot2 R package [41].

### 2.9. Phylogenetic Tree Analysis

BLASTP analysis of the candidate genes was performed against the Arabidopsis reference genome with an E-value threshold of 1 × 10^−5^, and hits with >50% sequence identity were retained to identify the Arabidopsis homologs [42]. Multiple sequence alignment was performed using MUSCLE [43], a maximum likelihood tree was constructed using FastTree [44], and the tree was formatted using ggtree [45].

### 2.10. qRT-PCR

Primers were designed using Premier 5.0, and their specificity was verified using the NCBI BLAST tool (https://www.ncbi.nlm.nih.gov, accessed 20 July 2026). At the three-leaf stage, rice seedlings were subjected to alkaline stress, and samples were collected at 0, 0.5, 1, 3, 7, and 14 d after treatment. Total RNA was extracted using the Ultra-Pure Total RNA Extraction Kit (Hangzhou Sumgen Biotechnology Co., Ltd., Hangzhou, China) and stored at −80 °C. First-strand cDNA was synthesized in a 10-μL reaction using the PrimeScript™ RT Master Mix (Takara Biomedical Technology (Beijing) Co., Ltd., Beijing, China) according to the manufacturer’s instructions. *OsActin* expression was used as an internal standard (Supplementary Table S1). qRT-PCR was performed on a LightCycler 96 system using SYBR Green (Vazyme Biotechnology Co., Ltd., Nanjing, China) as the fluorescent dye. Finally, the relative expression of the candidate genes was calculated using the 2^−∆∆CT^ method [46].

## 3. Results

### 3.1. Analysis of Phenotypic Traits

This study utilized the rice varieties QJ22 and Q74, obtained from the Qiqihar Branch of the Heilongjiang Academy of Agricultural Sciences, to investigate the genetic basis of rice tolerance to alkaline stress. Physiological and biochemical parameters were measured for both varieties after 14 days of growth under both normal and alkaline-stress conditions to elucidate the molecular mechanisms involved. Under normal conditions, there were no significant differences between the two varieties in any of the measured parameters. After alkaline stress treatment, QJ22 exhibited significantly greater plant height (Figure 1A,B), significantly higher chlorophyll content (Figure 1C), and significantly lower relative electrical conductivity (Figure 1D) than Q74. Additionally, the malondialdehyde (MDA) content of QJ22 was significantly lower than that of Q74 (Figure 1E), indicating less severe cell membrane damage. Meanwhile, the proline content, superoxide dismutase (SOD) activity, and peroxidase (POD) activity of QJ22 were all significantly higher than those of Q74 (Figure 1F–H), and it also exhibited a significantly lower Na^+^ content and a significantly higher K^+^ content (Figure 1I,J). Overall, under alkaline stress, QJ22 outperformed Q74 in terms of cell membrane stability, maintenance of photosynthetic pigments, antioxidant enzyme activity, and accumulation of osmoregulatory substances.

### 3.2. BSA Sequencing Analysis

To further identify alkaline-tolerance-related genes, we screened a segregating population derived from a cross between QJ22 and the alkaline-sensitive parent Q74 for alkaline stress phenotypes and selected extremely alkaline-tolerant individuals (tolerance pool, LA1–30) and extremely alkaline-sensitive individuals (sensitivity pool, LS1–30) (Supplementary Figure S1). BSA sequencing was then performed on the parental lines and the two extreme pools (Supplementary Table S2). Alignment of these sequences with the Oryza sativa subsp. japonica reference genome from the Ensembl Plants database revealed a total of 987,195 SNPs and 173,441 indels, which were reduced to 319,496 SNPs and 83,238 indels through trimming and filtering (Supplementary Table S3). We then selected the 402,734 high-quality SNPs and indels, which were homozygous in each parent but polymorphic between the two parents, for BSA analysis. Candidate regions associated with alkaline stress were identified using ΔSNP-index values (Figure 2A), Euclidean distance (ED) values (Figure 2B), and the G-matrix (Figure 2C). The results revealed a 2.25 Mb region on chromosome 10 between 11.65 Mb and 14.55 Mb (Supplementary Table S4). In summary, this 2.25 Mb region on chromosome 10 is a key candidate interval for the alkaline stress response in rice.

### 3.3. Transcriptome Analysis of Both Parents During the Seedling Stage Under Alkaline Stress

This study generated a total of 99.56 GB of RNA sequencing data, with an average of 8.30 GB per sample. After rigorous quality control, 95.51 GB of valid data were obtained, with an average of 7.96 GB per sample and GC content ranging from 50.92% to 52.76% (Supplementary Table S5), indicating that the sequencing data were of good quality. Additionally, PCA analysis showed clear separation of the two rice varieties along PC1 (34%) and the control/alkali treatments along PC2 (17.3%), with tight clustering of biological replicates, confirming the high reproducibility of the data (Supplementary Figure S2). Building on this, to characterize the alkali-stress response at the seedling stage, we performed comparative transcriptomic analysis of leaves from the alkali-tolerant parent QJ22 and the alkali-sensitive parent Q74 grown under normal conditions and subjected to 14 days of alkali stress. We identified 3555 differentially expressed genes (DEGs; 1733 upregulated, 1822 downregulated) in QJ22 and 3928 DEGs (1904 upregulated, 2024 downregulated) in Q74; in both parents, downregulated DEGs outnumbered upregulated ones. Among these, 2579 DEGs were shared by the two parental lines (Figure 3A–C, Supplementary Table S6). GO enrichment analysis revealed marked differences in the enriched GO terms between upregulated and downregulated genes in QJ22 and Q74. Among the upregulated genes, both parental lines were enriched in fundamental metabolic and epigenetic processes, including RNA modification, ncRNA metabolism, ribosome biogenesis, RNA processing, and methyltransferase activity; these genes were localized to the nucleolus and associated with cellular components such as the chloroplast envelope and thylakoid membranes. Notably, the upregulated genes in QJ22 were more strongly enriched in molecular functions such as misfolded protein binding, nuclease activity, and endonuclease activity (Figure 3D), whereas the upregulated genes in Q74 were also involved in biological processes such as chromatin organization and cold response, and were enriched in molecular functions including nuclease activity, transferase activity, and S-adenosylmethionine-dependent methyltransferase activity (Figure 3F). Among the downregulated genes, both parental lines showed suppression of components related to the photosynthetic system, thylakoids, and photosynthetic membranes, accompanied by reduced transmembrane transporter activity. In QJ22, downregulated genes were primarily enriched in leaf development, root development, and salt stress response; at the molecular level, they were associated with reduced transmembrane transporter activity for inorganic molecular entities, monoatomic cations, inorganic anions, and salts (Figure 3E). In contrast, downregulated genes in Q74 exhibited more pronounced stress-responsive characteristics, with significant enrichment in response to salt stress and response to water deprivation, and were associated with cellular components such as plant-type vacuoles and early endosomes; at the molecular level, in addition to reduced transmembrane transporter activity, they also showed altered activities of ubiquitin-like protein transferases and glycosyltransferases (Figure 3G).

### 3.4. Combined Analysis of BSA-Seq and RNA-Seq

BSA-Seq analysis identified a total of 157 candidate genes for alkaline stress response on chromosome 10 (Supplementary Table S7). A total of 2579 DEGs were shared between QJ22 and Q74 (Figure 3C). Through a combined analysis of BSA-Seq and RNA-Seq, we ultimately identified five candidate genes responsive to alkali stress: *Os10g0389000*, *Os10g0389500*, *Os10g0401000*, *Os10g0376200*, and *Os10g0390500* (Figure 4A,B). Further enrichment analysis of these candidate genes (Supplementary Figure S3) revealed that *Os10g0376200* was primarily enriched in cellular components such as the cytoplasm, axons, and cilia, as well as in biological processes including microtubule bundle formation and embryonic organ morphogenesis. *Os10g0390500* was specifically involved in molecular functions such as transaminase/transferase activity and pyridoxal phosphate binding, and in metabolic pathways including arginine biosynthesis and carbon fixation. *Os10g0389000* was primarily involved in the abscisic acid-mediated regulation of stomatal movement.

### 3.5. Analysis of Cis-Acting Regulatory Elements

Prediction and statistical analysis of *cis*-acting elements in the promoter regions (~2000 bp upstream) of the five candidate genes (Supplementary Table S8, Figure 5A) revealed that the types and numbers of elements varied considerably among the genes. Abscisic acid (ABA)-response elements were widely distributed across the five candidate genes, with the highest density observed in *Os10g0389500*, which harbored a total of 8 ABREs and 1 ABRE2. *Os10g0401000* contained 6 ABREs, whereas *Os10g0376200*, *Os10g0389000*, and *Os10g0390500* each carried 2–3 ABREs, together with varying numbers of ABA-related elements such as ABRE3a and ABRE4. The gibberellin response element P-box was detected only in *Os10g0389000* and *Os10g0401000*, whereas the ethylene response element ERE was present in the other four genes, being absent only in *Os10g0376200,* with the highest copy number (3) found in *Os10g0389000*. The salicylic acid response element as-1 was detected in all five genes; *Os10g0389000* contained four copies, more than any other gene. Regarding hormone element combinations, *Os10g0389000* was the only gene that carried all three types of hormone response elements—ABA (ABRE, ABRE3a, ABRE4), ethylene (ERE), and gibberellin (P-box)—and it also possessed a relatively high number of salicylic acid response elements (as-1). In addition, stress-related motifs in the *Os10g0389000* promoter—including two drought-induced MBS elements, one wound-response WUN motif, and three STRE elements—were relatively concentrated, and the number of MYC transcription factor binding sites (five) was also significantly higher than that in other genes (Figure 5B). The composition of the other genes varied: *Os10g0389500* contained a relatively large number of ABA-related elements and GC motifs, while *Os10g0401000* carried both P-box and TATC-box gibberellin-related elements. However, none of these genes exhibited the coexistence of multiple hormone-signaling elements in a concentrated manner. Overall, the *Os10g0389000* promoter displayed the most complex combination of hormone- and stress-signaling-related elements; this structural feature was consistent with its role in abscisic acid-mediated regulation of stomatal movement, as revealed by enrichment analysis.

### 3.6. Phylogenetic Tree Analysis

Phylogenetic analysis showed that the candidate genes and their Arabidopsis homologs clustered into three subclades. Group 3 contained the largest number of genes, and within its Subgroup V, Os10g0389000 was highly homologous to Arabidopsis *AtCML1* (*AT3G59450*), *AtCML19* (*AT4G37010*), and *AtCML20* (*AT3G50360*) (Figure 6). *AtCML20* downregulates the ABA signaling pathway by attenuating the response of guard cells to ABA, thereby inhibiting stomatal closure and reducing the plant’s sensitivity to ABA. When functionally absent, it enhances the ABA response, promoting the expression of stress-related genes and stomatal closure [47]. *Os10g0389000* is highly homologous to *AtCML20*, suggesting that *Os10g0389000* may play a similar role in abiotic stress responses in rice.

### 3.7. qRT-PCR

To further confirm whether *Os10g0389000* responds to alkaline stress, rice seedlings at the three-leaf-and-one-heart stage were treated with 75 mM NaHCO_3_ to impose alkaline stress. Leaves were collected at 0, 0.5, 1, 3, 7, and 14 days for real-time quantitative PCR (qRT-PCR) analysis. The results showed that under normal conditions (0 days), the expression levels of *Os10g0389000* were low in both the alkali-sensitive parent Q74 and the alkali-tolerant parent QJ22, with no significant difference between them (Figure 7). Following alkali stress treatment, the relative expression level of *Os10g0389000* in QJ22 increased rapidly, reaching 5.02 as early as 0.5 d and continuing to rise with stress duration, peaking at 17.46 by day 14. In contrast, the expression level in Q74 initially increased and then declined, peaking at 7.33 on day 7 and falling back to 5.33 by day 14. From 0.5 d onward, the expression level of Os10g0389000 in QJ22 was significantly higher than that in Q74 at all time points, indicating that *Os10g0389000* is strongly induced by alkaline stress and that its high expression is closely associated with alkaline tolerance.

## 4. Discussion

Soil salinization and alkalization severely affect seed germination, seedling growth, and crop yield, posing a serious threat to agricultural production. Therefore, enhancing rice tolerance to abiotic stress is of great practical importance. Under alkaline stress, lipid peroxidation occurs within plant cells, leading to the massive accumulation of reactive oxygen species (ROS), such as hydrogen peroxide, and malondialdehyde (MDA) [48]. Additionally, alkali stress increases cell membrane permeability and relative electrical conductivity by causing membrane lipid peroxidation [49]. Furthermore, the accumulation of salts in leaves under alkali stress reduces chlorophyll content and inhibits photosynthesis [50]. Previous studies have shown that elevated levels of organic osmolytes, such as trehalose, proline, and betaine, enhance plant tolerance to abiotic stress, while increased antioxidant enzyme activity effectively removes the products of oxidative damage [51,52]. In this study, QJ22 exhibited greater cell membrane stability, higher chlorophyll content, higher proline accumulation, and higher antioxidant enzyme activity under alkaline stress compared to Q74 (Figure 1C–H). Consequently, QJ22 showed greater tolerance to alkaline stress than Q74.

Traditional map-based cloning techniques often face challenges in constructing high-density maps and near-isogenic lines, whereas BSA-Seq can rapidly identify major-effect loci and has become an important tool for dissecting crop responses to abiotic stress [53]. However, this method typically identifies hundreds of candidate genes within a single mapping interval, making it difficult to pinpoint the causal gene(s) [54]. Therefore, combining BSA-Seq with RNA-Seq and leveraging transcriptomic differential expression data to filter genes within the mapped regions can effectively narrow the candidate list, representing a viable strategy. In this study, we used BSA-Seq and RNA-Seq to identify five candidate genes for alkaline tolerance on rice chromosome 10 and performed GO and KEGG enrichment analyses (Figure 3A,B). These five genes were found to play diverse roles; for example, *Os10g0376200* is involved in microtubule bundle formation and embryonic organ morphogenesis, and *Os10g0390500* is involved in transaminase activity, arginine biosynthesis, and carbon fixation pathways. This suggests that different genes within this region function synergistically and complementarily at the structural support–metabolic supply level (Supplementary Figure S3). Notably, *Os10g0389000* plays a particularly critical role in this process: it is involved in the ABA-mediated regulation of stomatal movement, and ABA is a key signaling molecule in plant abiotic stress responses. Under alkaline stress, the root system first perceives the high-pH environment and, through ABA signaling, rapidly transmits this signal to the leaves, where it regulates stomatal closure to reduce transpiration and passive ion uptake. This response occurs prior to cytoskeletal reorganization and metabolic adjustments and thus serves as an upstream switch in the stress response [55]. Compared with *Os10g0376200* and *Os10g0390500*, which primarily fulfill structural maintenance and metabolic precursor supply functions, respectively, *Os10g0389000* directly mediates the initial perception of stress and the initiation of the response, thereby determining whether a cascade of downstream defense measures can be rapidly mobilized. Therefore, *Os10g0389000* may act as a central regulatory node in this alkali-tolerance network, and genetic variations or changes in its expression could play a decisive role in the alkali-tolerance phenotype conferred by this genomic region.

CREs play a key role in regulating the transcription of genes involved in abiotic stress responses [56]. The ABRE is a core *cis*-acting element for ABA-dependent gene expression; in Arabidopsis, the bZIP1 transcription factor regulates ABA signaling by binding to the ABRE, thereby enhancing plant tolerance to abiotic stress [57]. As a key regulatory element of gibberellin (GA) signaling, the P-box plays a crucial role in plant responses to abiotic stress. The *MYB12* transcription factor regulates GA biosynthesis and signal transduction by binding to the P-box *cis*-acting element in the promoters of GA biosynthesis genes, thereby influencing Arabidopsis growth and development [58,59]. Ethylene plays an indispensable role in regulating plant growth and stress responses, with the ERE element serving as a key *cis*-acting node in ethylene-mediated abiotic stress signaling [60]. Under conditions such as drought, high salinity, and waterlogging, the ethylene signaling pathway is activated and triggers a cascade that culminates in the specific binding of Ethylene Response Factor (*ERF*) transcription factors to ERE elements in the promoters of downstream stress-responsive genes. This binding initiates target gene transcription and thereby enhances plant stress tolerance [61,62,63]. In Arabidopsis, salicylic acid (SA) enhances the binding of *TGA* transcription factors (such as *TGA2/3*) to the as-1 cis-acting element via the signal regulator *NPR1*, thereby activating the transcription of downstream defense genes such as *PR-1*, forming the core transcriptional regulatory module of SA signaling [64,65]. In this study, among the five candidate genes, the promoter region of *Os10g0389000* not only contained three types of hormone response elements—ABA (ABRE, ABRE3a, ABRE4), ethylene (ERE), and gibberellin (P-box)—but also harbored a large number of salicylic acid response elements (as-1), along with an enrichment of drought- and wounding-related stress motifs (Figure 5A,B). The concentrated distribution of these multi-hormone signaling elements suggests that *Os10g0389000* may coordinate stomatal movement under abiotic stress by integrating multiple signaling pathways, including ABA, ET, GA, and SA, which is highly consistent with its functional enrichment in ABA-mediated regulation of stomatal movement. In contrast, although the other genes contain certain response elements, they lack such a complex multi-hormone synergistic module, suggesting that Os10g0389000 may serve as the central hub for signal integration in this gene cluster. *AtCML20* is a member of the *CML* family. As a calcium-binding negative regulator, *AtCML20* acts in guard cells by inhibiting ABA-activated S-type anion currents and relieving the inhibition of inward K^+^ currents, thereby attenuating ABA-induced stomatal closure and negatively regulating drought tolerance in Arabidopsis [47,66,67]. *Os10g0389000* is also a member of the *CML* family and is highly homologous to *AtCML20*, suggesting that *Os10g0389000* may have a similar function (Figure 6). qRT-PCR analysis revealed that *Os10g0389000* was rapidly and persistently induced under alkaline stress, and the magnitude of its upregulation was significantly greater in the alkali-tolerant variety QJ22 than in the sensitive variety Q74 (Figure 7). This differential expression pattern between varieties is consistent with the enrichment of multi-hormone signaling elements in its promoter, supporting the hypothesis that *Os10g0389000* may contribute to rice alkali tolerance through a complex transcriptional regulatory network. Nevertheless, its exact function and regulatory mechanisms await elucidation through future transgenic functional validation and protein biochemical experiments.

## 5. Conclusions

This study systematically analyzed the phenotypic differences and genetic effects between the alkali-tolerant variety QJ22 and the sensitive variety Q74. Through combined BSA-Seq and RNA-Seq analysis, five candidate genes involved in the alkali stress response were identified. *Cis*-acting element prediction, phylogenetic tree analysis, and qRT-PCR results indicated that *Os10g0389000* is the key gene responsible for the differences in alkali tolerance between the two varieties. The QTLs identified using the mapping strategy employed in this study are expected to be effectively applied in future alkali-tolerance breeding, holding significant theoretical and practical value for elucidating the physiological and molecular mechanisms of rice alkali tolerance and for advancing genetic improvement and the development of salt- and alkali-tolerant varieties.

## Figures and Tables

**Figure 1:**
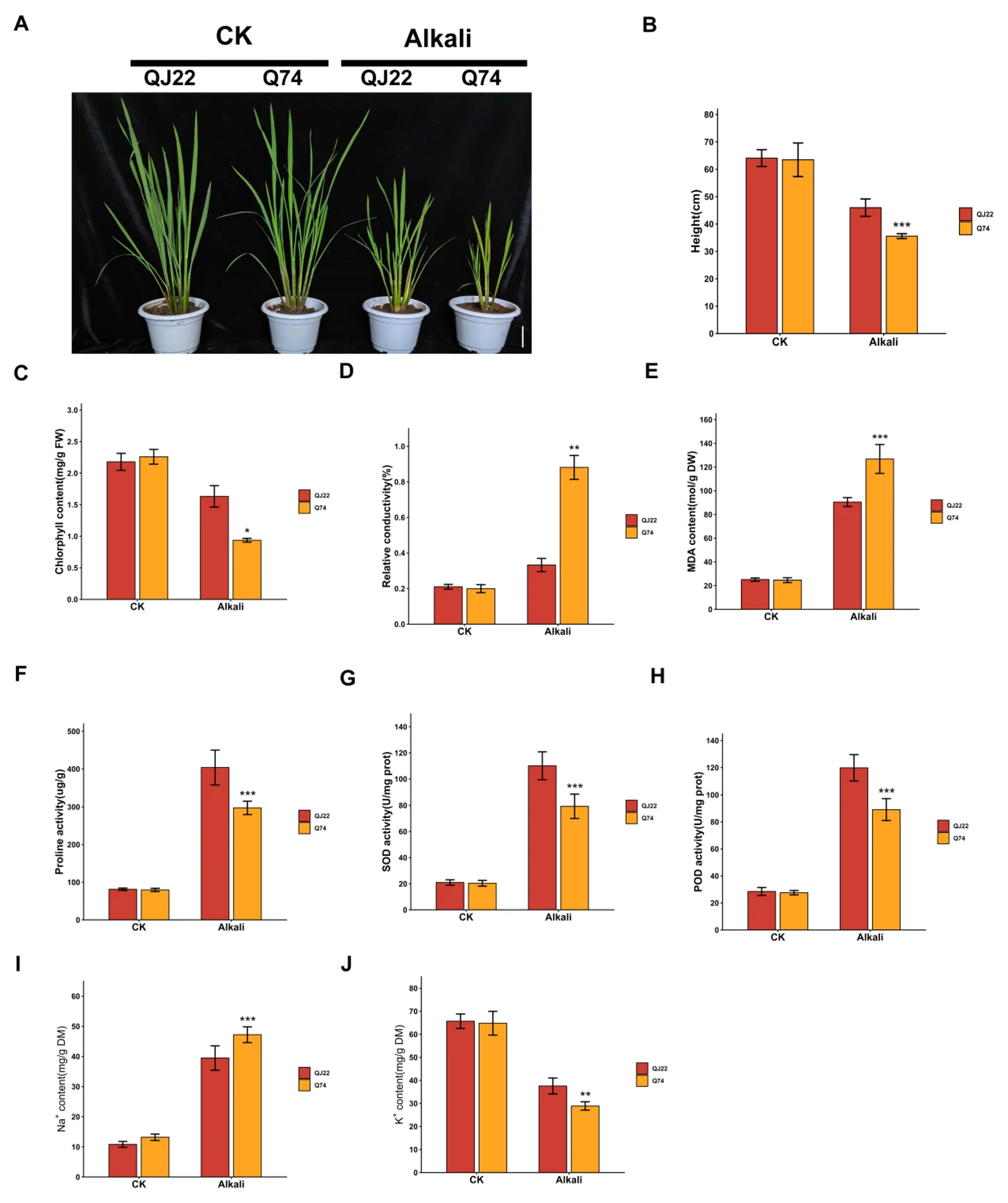
Phenotypic comparison of QJ22 and Q74 plants under normal and alkaline stress conditions for 14 days. (**A**) Phenotypes of the two varieties after 14 days of growth under normal and alkaline-stress conditions. (**B**–**J**) Comparison of (**B**) plant height, (**C**) chlorophyll content, (**D**) relative electrical conductivity, (**E**) MDA content, (**F**) proline content, (**G**) SOD activity, (**H**) POD activity, (**I**) Na^+^ content, and (**J**) K^+^ content between QJ22 and Q74 following 14 days of treatment with 75 mM NaHCO_3_. Data are presented as the mean ± standard deviation of three independent replicates. Asterisks indicate statistically significant differences between the two varieties as determined by Student’s *t*-test (* *p* < 0.05, ** *p* < 0.01, *** *p* < 0.001).

**Figure 2:**
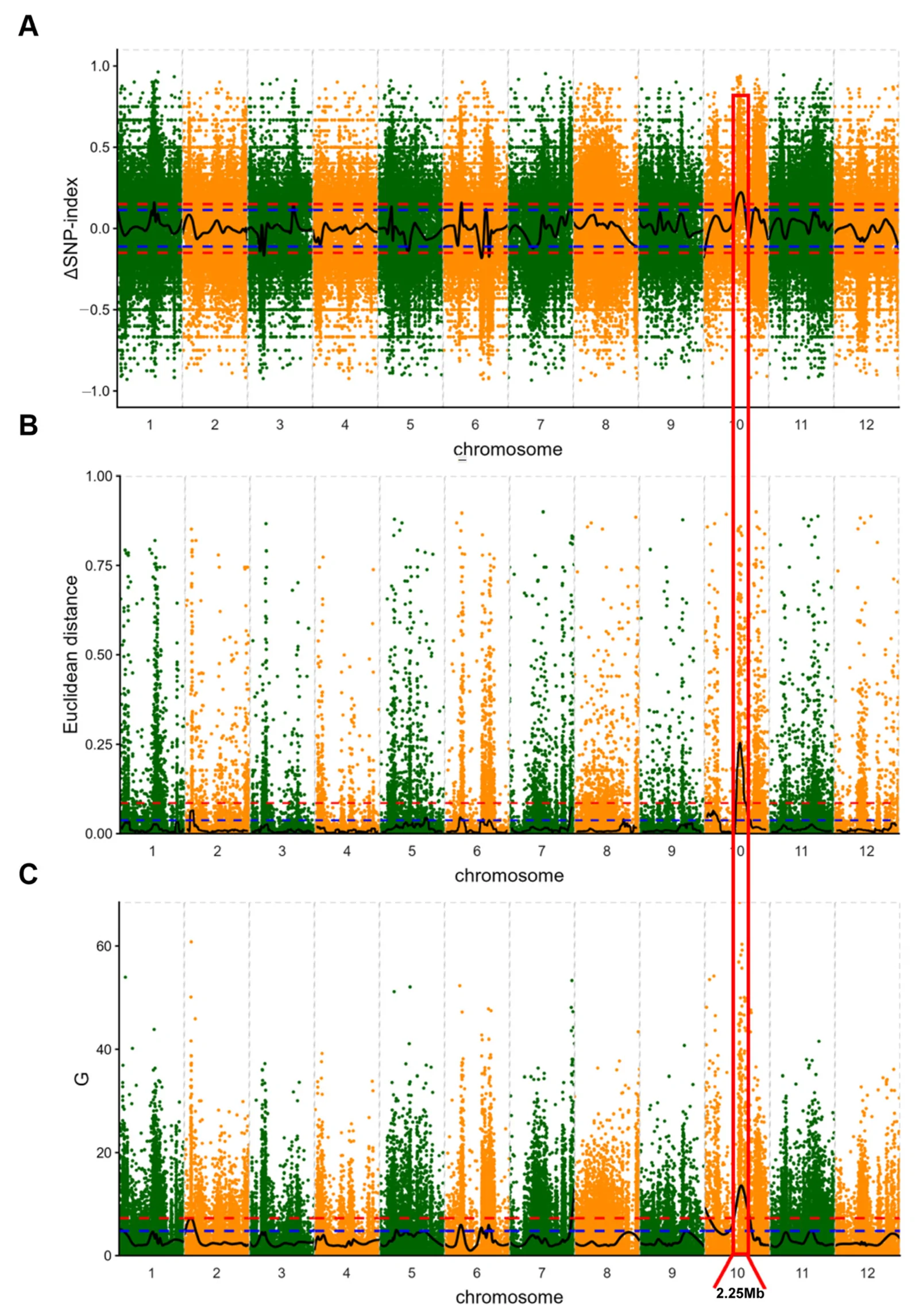
QTL-seq analysis based on BSA sequencing under alkaline stress. (**A**) Manhattan plot showing the distribution of the ΔSNP-index across chromosomes. (**B**) Manhattan plot showing the distribution of Euclidean distance (ED) across chromosomes. (**C**) Manhattan plot showing the distribution of the G-statistic across chromosomes. The blue and red lines represent the 95% and 99% confidence intervals, respectively, while the black line represents the mean of the three algorithms; these means were plotted using a sliding-window analysis. The numbers on the x-axis represent.

**Figure 3:**
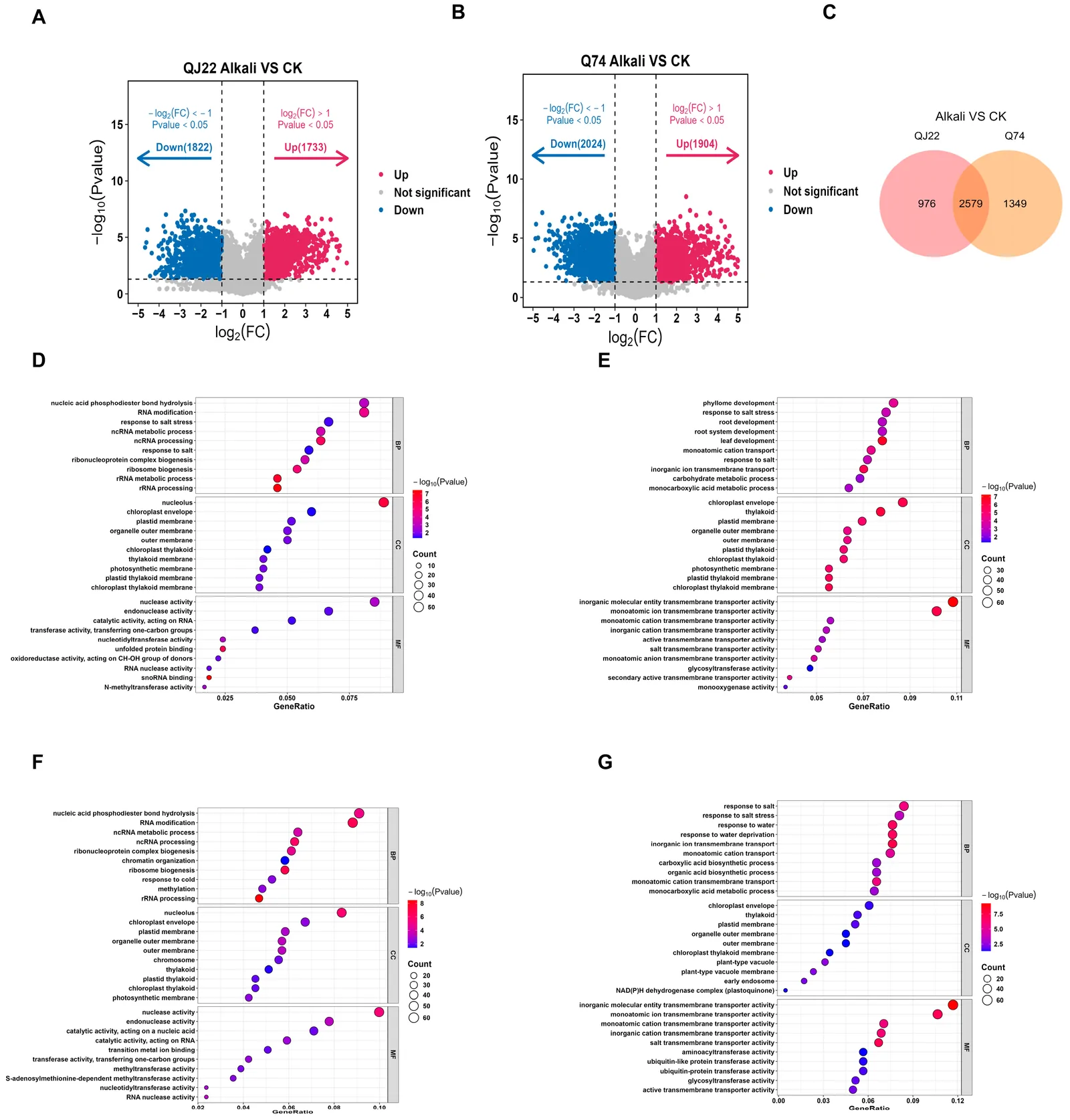
Differentially expressed genes (DEGs) in the seedling stage of QJ22 and Q74. (**A**) DEGs in QJ22 under alkaline stress. (**B**) DEGs in Q74 under alkaline stress. (**C**) Overlap of DEGs between QJ22 and Q74. (**D**,**E**) GO enrichment analysis of upregulated and downregulated DEGs in QJ22. (**F**,**G**) GO enrichment analysis of upregulated and downregulated DEGs in Q74. (*p* < 0.05). In the volcano plots, blue indicates downregulated DEGs and red indicates upregulated DEGs.

**Figure 4:**
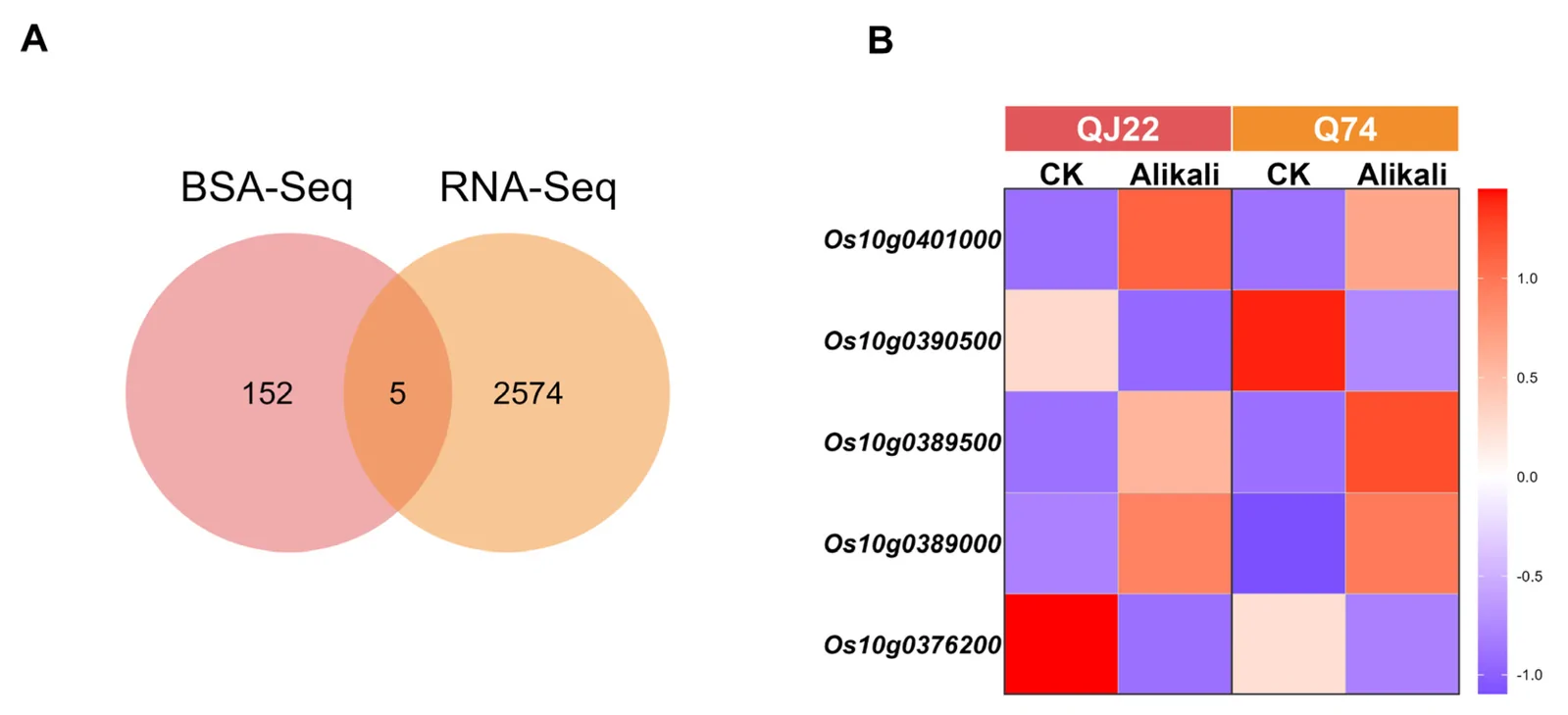
Combined BSA-Seq and RNA-Seq analysis. (**A**) Overlap between the DEGs identified in QJ22 and Q74 and the BSA-Seq candidate genes. (**B**) Heatmaps showing the expression levels of the five candidate genes in QJ22 and Q74. Blue, downregulation; red, upregulation.

**Figure 5:**
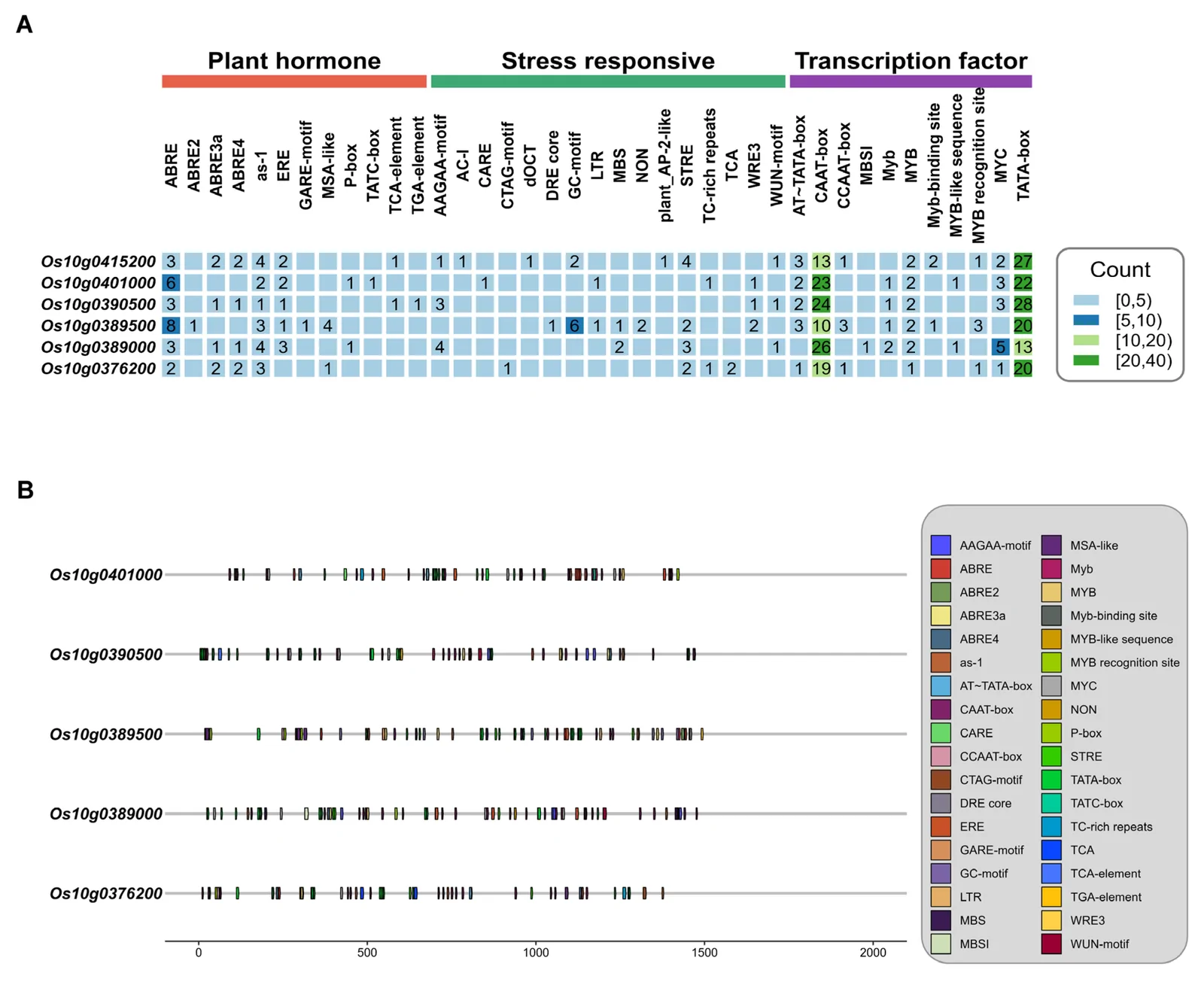
Analysis of 2-kb *cis*-acting elements in candidate genes. (**A**) Number of *cis*-acting elements in candidate genes. (**B**) Distribution of *cis*-acting elements in candidate genes.

**Figure 6:**
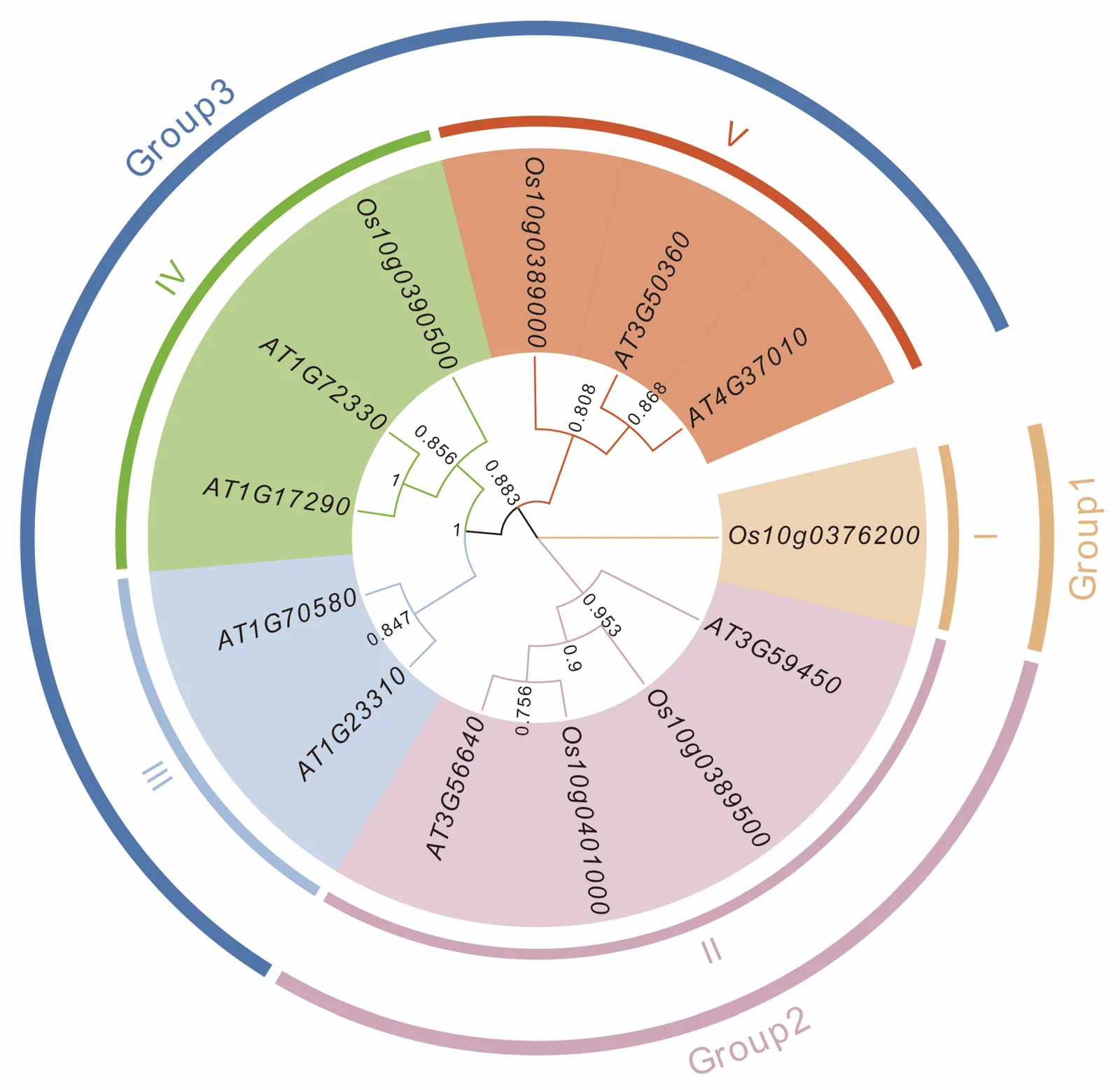
Phylogenetic tree of the candidate genes and their Arabidopsis homologs. The outer ring designations (Group1, Group2, Group3) represent three major phylogenetic clades, while the inner Roman numerals (I–V) denote the corresponding sub-clades within these groups. Node values represent statistical confidence (bootstrap values).

**Figure 7:**
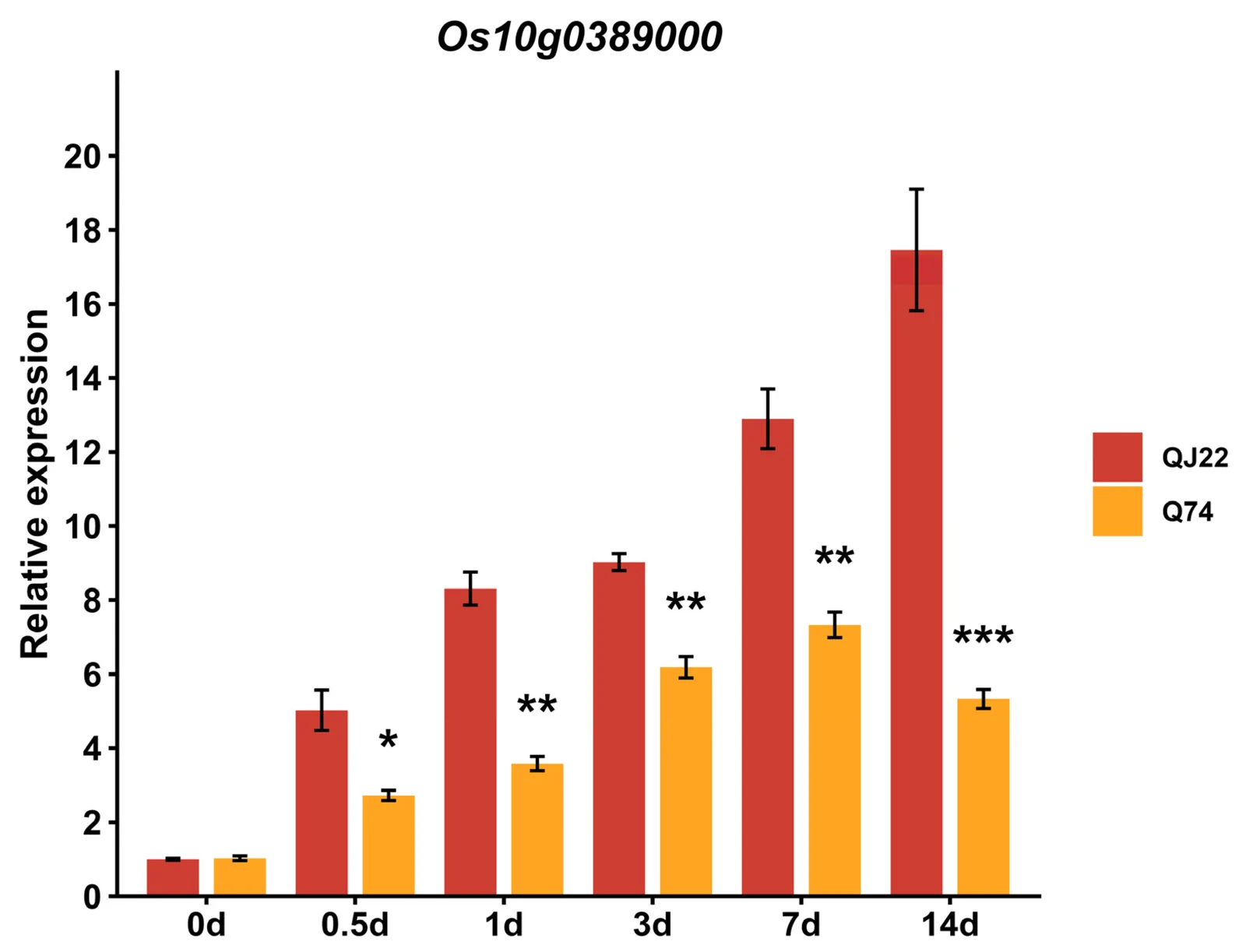
Relative expression levels of Os10g0389000 in QJ22 and Q74 under alkali stress. Error bars represent SD (*n* = 3). Significant differences between the two lines at each time point were determined by Student’s *t*-test (* *p* < 0.05, ** *p* < 0.01, *** *p* < 0.001).

## Data Availability

The original contributions presented in the study are included in the article; further inquiries can be directed to the corresponding authors.

## Supplementary Materials

The following supporting information can be downloaded at https://www.mdpi.com/article/10.3390/genes17101162/s1. Supplementary Figure S1: Kernel density plot of plant height in the F_2_ population under alkaline stress; Supplementary Figure S2: Principal component analysis (PCA) of transcriptome data in rice varieties QJ22 and Q74 under control and alkali stress; Supplementary Figure S3: GO and KEGG enrichment analysis of candidate genes; Supplementary Table S1: Sequence of primers used in qRT-PCR; Supplementary Table S2: BSA-Seq quality control results; Supplementary Table S3: Number of SNPs and indexs; Supplementary Table S4: Information on BSA-Seq co-localized gene regions; Supplementary Table S5: Transcriptome sequencing quality control information; Supplementary Table S6: Gene information for the BSA-mapped region; Supplementary Table S7: Gene information for the BSA-mapped region; Supplementary Table S8: Information on *cis*-acting elements of candidate genes.
